# Combination antiretroviral therapy (cART) restores HIV-1 infection-mediated impairment of JAK-STAT signaling pathway

**DOI:** 10.18632/oncotarget.15121

**Published:** 2017-02-06

**Authors:** Man-Qing Liu, Min Zhao, Wen-Hua Kong, Li Tang, Fang Wang, Ze-Rong Zhu, Xia Wang, Hong-Yan Qiu, Dun-Jin Zhou, Xu Wang, Wen-Zhe Ho, Wang Zhou

**Affiliations:** ^1^ Wuhan Centers for Disease Prevention and Control, Wuhan 430015, China; ^2^ Wuhan Institute of Dermatology and Venereology, Wuhan 430030, China; ^3^ Department of Pathology and Laboratory Medicine, Temple University Lewis Katz School of Medicine, Philadelphia, PA 19104, USA

**Keywords:** JAK-STAT signaling pathway, HIV-1, combination antiretroviral therapy (cART), peripheral blood mononuclear cells (PBMCs)

## Abstract

JAK-STAT signaling pathway has a crucial role in host innate immunity against viral infections, including HIV-1. We therefore examined the impact of HIV-1 infection and combination antiretroviral therapy (cART) on JAK-STAT signaling pathway. Compared to age-matched healthy donors (*n* = 18), HIV-1-infected subjects (*n* = 18) prior to cART had significantly lower expression of toll-like receptors (TLR-1/4/6/7/8/9), the IFN regulatory factors (IRF-3/7/9), and the antiviral factors (OAS-1, MxA, A3G, PKR, and Tetherin). Three months’ cART partially restores the impaired functions of JAK-STAT-mediated antiviral immunity. We also found most factors had significantly positive correlations (*p* < 0.05) between each two factors in JAK-STAT pathway in healthy donors (98.25%, 168/171), but such significant positive associations were only found in small part of HIV-1-infected subjects (43.86%, 75/171), and stably increased during the cART (57.31%, 98/171 after 6 months’ cART). With regard to the restoration of some HIV-1 restriction factors, HIV-1-infected subjects who had CD4+ T cell counts > 350//μl responded better to cART than those with the counts < 350/μl. These findings indicate that the impairment of JAK-STAT pathway may play a role in the immunopathogenesis of HIV-1 disease.

## INTRODUCTION

Innate immunity plays a critical role in host defense mechanisms against viral infections, including HIV-1 [1]. The innate immune system includes three classes of receptors, Toll-like receptors (TLRs), retinoic acid-inducible gene I (RIG-I)-like receptors (RLRs), and nucleotide oligomerization domain (NOD)-like receptors (NLRs) [2]. Among these receptors, TLRs function as the primary sensors of pathogens, activation of which can stimulate several signaling pathways [3], including the Janus kinase–signal transducer and activator of transcription (JAK-STAT).

JAK-STAT signaling pathway is of importance in interferon (IFN)-mediated antiviral activities [4]. The IFN-stimulated gene factor 3 (ISGF3), formed by STAT1, STAT2, and interferon regulatory factor (IRF)-9, is a pivotal transcription factor in the JAK-STAT pathway [4–6]. The production of IFNs in cells is in response to the infection by a variety of viruses [6], which mainly recognized the presence of the viral genome or viral proteins by TLRs [3, 7–9]. There are 13 TLRs identified in mice and 11 in humans, of which TLR-3, −7, −8, and −9 can detect viral or bacterial nucleic acids [3], and TLR-1 and 6 can recognize some viral proteins [10]. All TLRs except TLR-3 have been found to be able to activate a common signaling pathway leading to the production of the proinflammatory cytokines via myeloid differentiation factor 88 (MyD88) [2]. TLR activation induces signaling cascades that mainly involve various IRFs [6], in which IRF3 and IRF7 played essential roles in virus-induced transcriptional activation of IFN-β [6, 11]. The phosphorylation of IRFs results in the induction of antiviral factors such as the IFN-stimulated gene 56 (ISG-56), myxovirus resistance-1 (MxA), 2′,5′-oligoadenylate synthetase I (OAS-1), and tetherin [12–14]. By blocking IFN signal transduction, including inhibition of phosphorylation [15] or nuclear translocation [16] of STAT1, STAT2, STAT3, or upregulation of SOCS [17, 18], virus can escape from the powerful antiviral effects of the IFN system [6, 19, 20].

It has been documented that HIV-1 infection could immediately trigger strong innate and adaptive immune responses [21]. *In vitro* studies revealed that IFN-λ can upregulate cellular anti-HIV-1 factors through JAK-STAT signaling pathway and significantly inhibited HIV-1 infection [1, 12]; TLR-3 activated by Poly I:C can induce multiple anti-HIV-1 factors and inhibit HIV-1 infection of macrophages [22]. These *in vitro* findings indicate that host innate immunity is critical in restricting HIV-1 replication and spread. However, by interfering with the signaling moleculars through its proteins, HIV-1 can suppress the induction of IFNs and antiviral ISGs and persist in immune cells. Our previous *in vitro* study [12] demonstrated that JAK-STAT pathway is involved in the induction of the anti-HIV-1 cellular factors and HIV-1 inhibition in macrophages [23]. Thus, it is of importance to determine whether HIV-1 infection impairs the JAK-STAT signaling pathway and whether cART can reverse HIV-1 infection-mediated injury of JAK-STAT pathway.

## RESULTS

### Subject information

Eighteen HIV-1-infected subjects had CD4+ T cell counts of 62~670 /μL (mean of 362) and viral loads of 0.081~79 × 10e4 copies/mL (mean of 6.9 × 10e4) at the time of study enrollment (Table 1). They also had normal aspartate transaminase (AST, 27.4 ± 18.7 IU/mL) and alanine transaminase (ALT, 26.6 ± 16.1 IU/mL), and were satisfied for antiviral treatment. The majority (17 out of 18) of HIV-1-infected subjects were men who have sex with men (MSM) and treated with TDF+3TC+EFV. During the course of study, neither liver injury (AST < 72 IU/L) nor clinical symptoms were reported in these study subjects. Age-matched healthy donors (*n* = 18) were enrolled as the control subjects.

**Table 1:** Demographic and clinical characteristics of HIV-1-infected subjects and control subjects

Category	HIV-1-infected subjects (*n* = 18)	Control subjects (*n* = 18)
Age (Years)*	30.33 ± 6.23	26.44 ± 8.39
Range of age (Years)	22–44	21–47
Gender (male/female)	17/1	16/2
**CD4+ T-cell counts (cells/μL)**		
Prior to cART	362 ± 163	-
1 month cART	441 ± 169	-
3 months cART	444 ± 192	-
6 months cART	460 ± 199	-
**HIV-1 load (copies/mL)**		
pre-cART	69184 ± 184778	-
1 month cART	167± 286	-
3 months cART	36 ± 57	-
6 months cART	28 ± 70	-
**Combination antiretroviral therapy (cART)**		
TDF+3TC+EFV	17	-
AZT+3TC+NVF	1	-

Data was expressed as mean ± SD.

*There was no age difference between two groups (*p* = 0.1236).

### Effect of HIV-1 infection or cART on TLRs and IRFs

**Figure 1:**
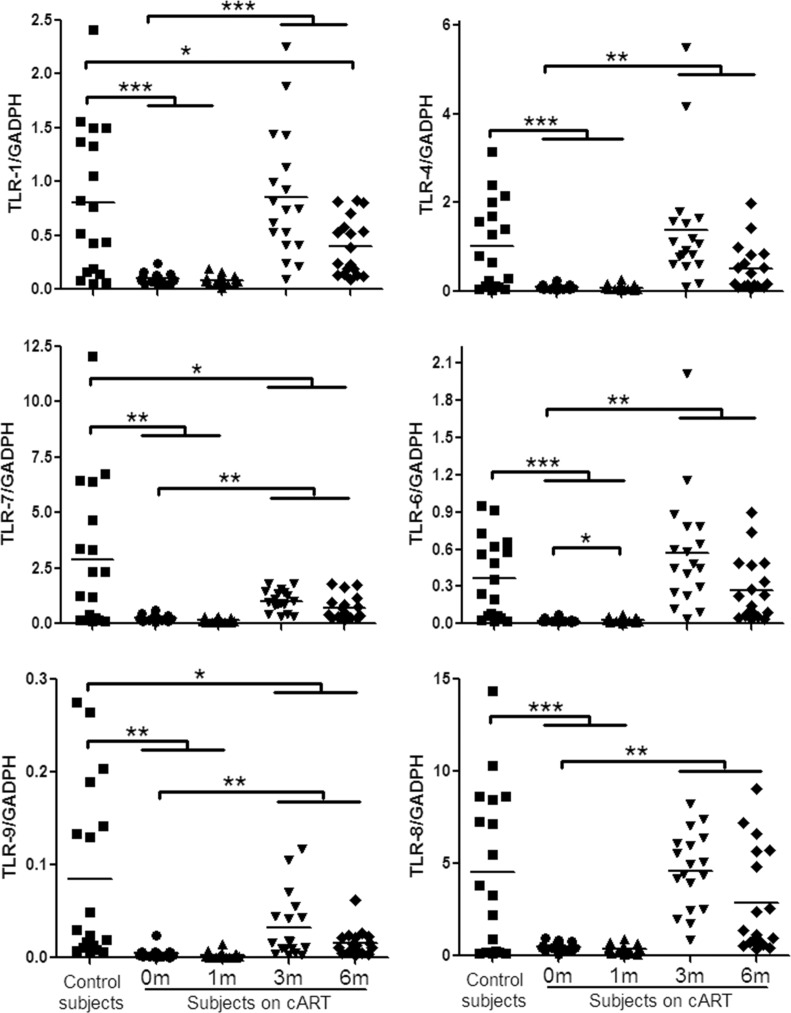
TLRs expression in PBMCs of HIV-1-infected subjects on cART PBMCs were collected from HIV-1-infected subjects (*n* = 18) prior to (0 m) or 1 month (1 m), 3 months (3 m), and 6 months (6 m) after cART. Age-matched healthy subjects (*n* = 18) were used as a control group. Cellular RNAs extracted from PBMCs were subjected to quantitative PCR for the indicated TLRs. The expression levels were shown relative to glyceraldehyde-3-phosphate dehydrogenase (GAPDH), and the significant differences from paired or unpaired *t* tests were shown by *p < 0.05, **p < 0.01, and ***p < 0.001.

**Figure 2:**
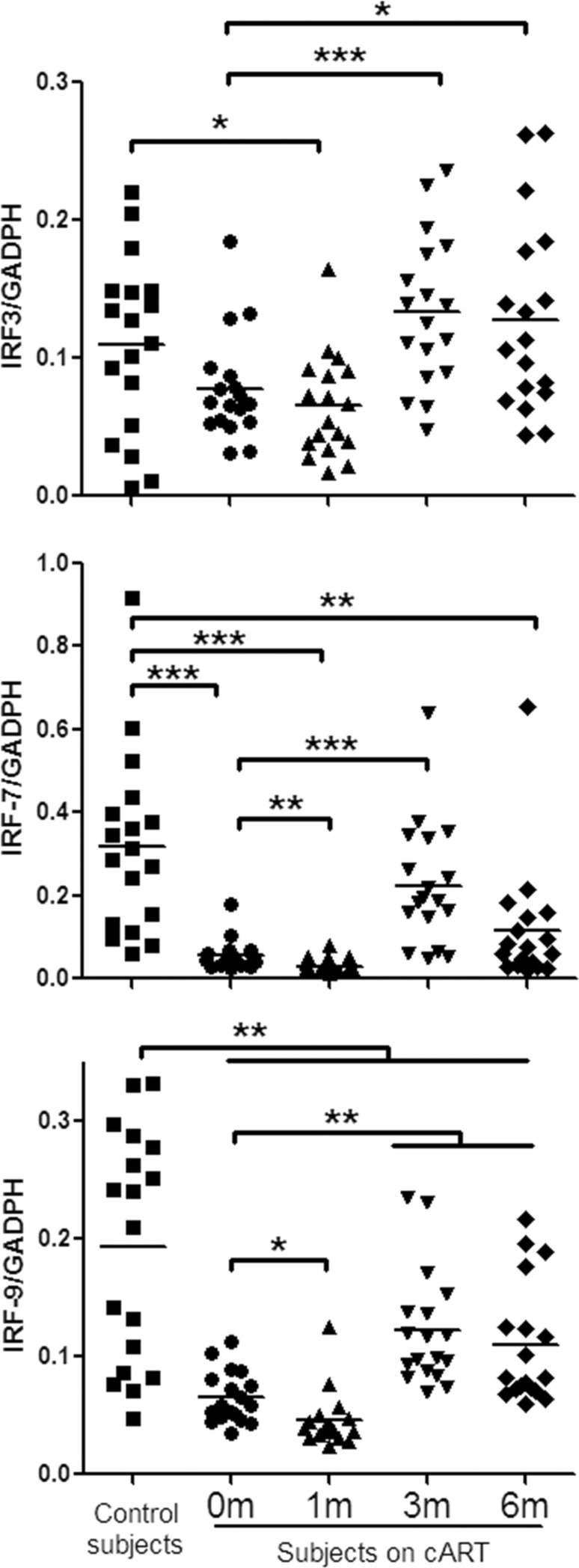
IFN regulatory factor (IRF)-3/7/9 expression in PBMCs of HIV-1-infected subjects on cART PBMCs were collected from HIV-1-infected subjects (*n* = 18) prior to (0 m) or 1 month (1 m), 3 months (3 m), and 6 months (6 m) after cART. Age-matched healthy subjects (*n* = 18) were set as a control group. Cellular RNAs extracted from PBMCs were subjected to quantitative PCR for the indicated IRFs. The expression levels were shown relative to glyceraldehyde-3-phosphate dehydrogenase (GAPDH), and the significant differences from paired or unpaired *t* tests were shown by *p < 0.05, **p < 0.01, and ***p < 0.001.

TLRs can recognize viral RNA or DNA and initiate antiviral signaling pathways [7, 12, 24]. We thus examined six TLRs (TLR-1/4/6/7/8/9) that are known to be involved in antiviral immunity [9, 24, 25]. Figure 1 showed that peripheral blood mononuclear cells (PBMCs) from HIV-1-infected subjects before or one month after cART had lower levels of the TLRs than those of the control subjects. Three-month cART could reverse the levels of the TLRs. However, these levels of TLRs were still lower than those of uninfected subjects. We next analyzed the expression of three IFN regulatory factors (IRF-3, IRF-7 and IRF-9) that have critical role in the regulation of IFN. As shown in Figure 2, HIV-1-infected subjects had significant lower levels of IRF-7 and IRF-9 than the control subjects. Three-month treatment with cART could restore the levels of these IRFs (Figure 2).

### Impact of HIV-1 infection or cART on anti-HIV-1 factors

**Figure 3:**
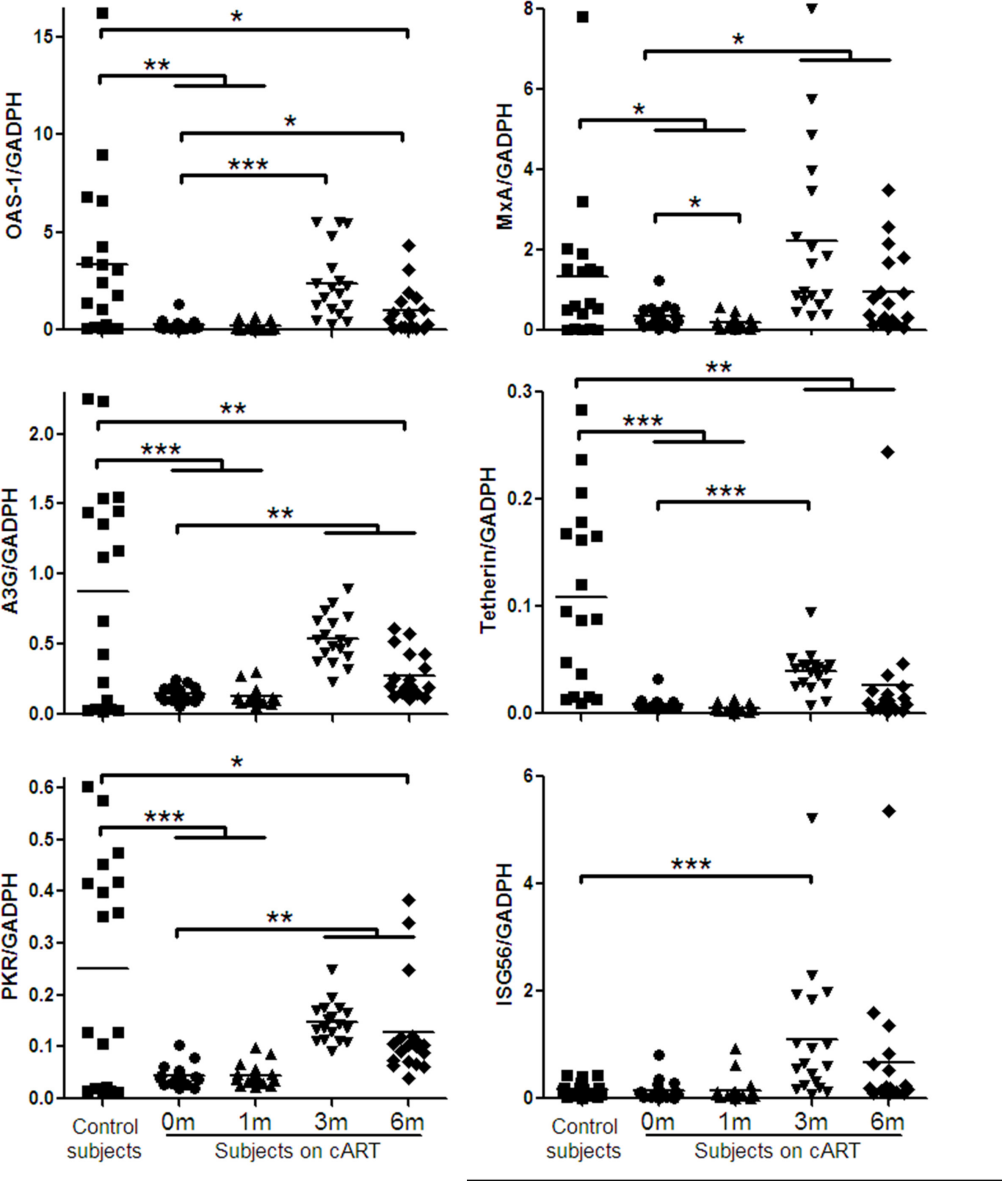
Cellular anti-HIV-1 factors expression in PBMCs of HIV-1-infected subjects on cART PBMCs were collected from HIV-1-infected subjects (*n* = 18) prior to (0 m) or 1 month (1 m), 3 months (3 m), and 6 months (6 m) after cART. Age-matched healthy subjects (*n* = 18) were used as a control group. Cellular RNAs extracted from PBMCs were subjected to quantitative PCR for anti-HIV-1 factors (OAS-1, MxA, A3G, Tetherin, PKR, ISG56). The expression levels were shown relative to glyceraldehyde-3-phosphate dehydrogenase (GAPDH), and the significant differences from paired or unpaired *t* tests were shown by *p < 0.05, **p < 0.01, and ***p < 0.001.

Several cellular factors (OAS-1, MxA, APOBEC3G (A3G), Tetherin, protein kinase (PKR), and interferon-stimulated gene 56 (ISG56)) in JAK-STAT pathway have been shown to have anti-HIV-1 activities. We thus examined the expression of these factors in PBMCs of the study subjects. As shown in Figure 3, HIV-1-infected subjects had significantly lower levels of the HIV-1 restriction factors than the control subjects. Three-month cART could significantly increase the levels of these factors in PBMCs of HIV-1-infected subjects (Figure 3). Comparing with HIV-1-infected subjects at prior to or after cART (1, 3 or 6 months), uninfected subjects had the highest levels of the HIV-1 restriction factors (Figure 3).

### Correlation analysis on cellular factors in JAK-STAT pathway

**Figure 4:**
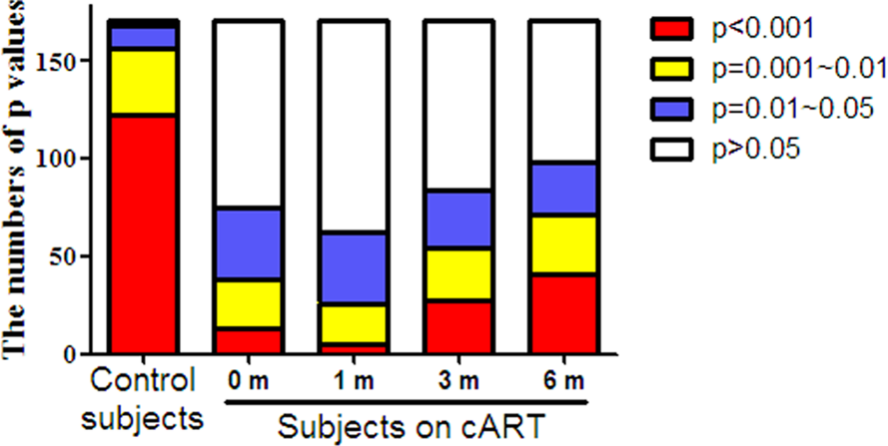
The distribution of *P* values for the correlations between each two factors in JAK-STAT pathway in PBMCs of HIV-1-infected subjects on cART PBMCs were collected from HIV-1-infected subjects (*n* = 18) prior to (0 m) or 1 month (1 m), 3 months (3 m), and 6 months (6 m) after cART. Age-matched healthy subjects (*n* = 18) were set as a control group. Cellular RNAs extracted from PBMCs were subjected to quantitative PCR for the factors in JAK-STAT pathway, including TLRs (TLR-1, TLR-4, TLR-6, TLR-7, TLR-8, TLR-9), IRFs (IRF-3, IRF-7, IRF-9), antiviral factors (OAS-1, MxA, A3G, PKR, ISG-56, Tetherin), inflammatory cytokine (IL-6, IL-10), and adaptor molecular (MyD88, STAT-2). Supplementary Table 1 showed the detailed significances of correlations between each two factors by Spearman. The results in the figure are expressed as the numbers at four significant stages (*p* < 0.001, *p* = 0.001~0.01, *p* = 0.01~0.05, *p* > 0.05).

Our results indicated similar changes of TLRs, IRFs, and cellular anti-HIV-1 factors in HIV-1-infected subjects, especially during the cART, thus, we supposed all factors in JAK-STAT pathway should have positive corrections between each two factors. To clarify this issue, the correlations between each two factors in JAK-STAT pathway were determined. Among 19 tested molecular (TLR-1, TLR-4, TLR-6, TLR-7, TLR-8, TLR-9, IRF-3, IRF-7, IRF-9, OAS-1, MxA, A3G, PKR, ISG-56, Tetherin, IL-6, IL-10, MyD88, STAT-2), 171 correlations were calculated in total. In healthy donors, almost of all correlations (98.25%, 168/171) were positive significance (*p* < 0.05), and most (72.62%, 122/168) of significances were < 0.001 (Figure 4 and Supplementary Table 1). However, only small part of significant correlations (*p* < 0.05) were found in HIV-1-infected subjects (43.86%, 75/171) prior to cART, and most of which belonged to 0.01~0.05 (49.33%, 37/75). During the cART, the numbers of significant correlations stably increased to 98 at six months, but remained less than that of the control subjects.

### The association to CD4+ T cells counts or HIV-1 loads

**Figure 5:**
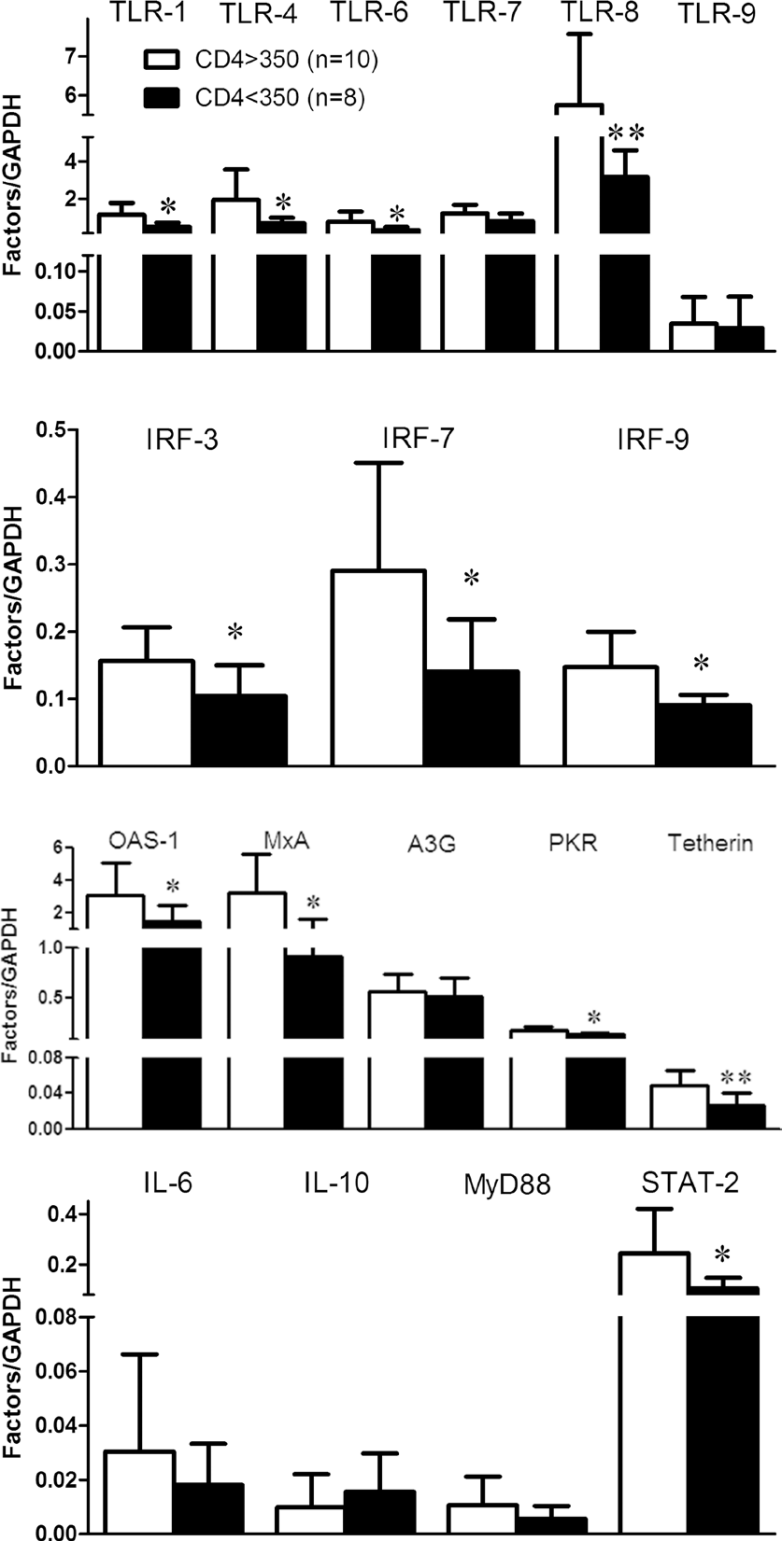
The response to cART for the factors in JAK-STAT pathway in PBMCs of HIV-1-infected subjects PBMCs were collected from two groups of HIV-1-infected subjects: CD4 > 350 cells/μL (*n* = 10) and CD4 < 350 cells/μL (*n* = 8) prior to cART. After three months’ cART, cellular RNAs extracted from PBMCs were subjected to quantitative PCR for the factors in JAK-STAT pathway. Error bars represent SD, with significance from *t* test shown by *p < 0.05 and **p < 0.01.

To explore the association of JAK-STAT pathway with disease process, we further examined the correlation of cellular factors in JAK-STAT pathway with CD4+ T cells and plasma viral loads. CD4+ T cells counts were increased to 444 ± 192 /μL after three-month cART and to 460 ± 199/μL after six-month cART as compared to 362 ± 163/μL prior to cART (Table 1). In contrast, HIV-1 loads were decreased to 36 ± 57 copies/mL 3 months after cART and to 28 ± 70 copies/mL after six-month cART, as compared to 69184 ± 184778 copies/mL prior to cART (Table 1). Although no significant correlation was found between antiviral factors and CD4+ T cell counts or HIV-1 loads in the study subjects prior to or after cART, the subjects with > 350/μl CD4+ T cells counts showed better response (*p* < 0.05) to cART after three-months’ treatment (Figure 5). Specifically, after three-months’ antiviral therapy, the HIV-1-infected subjects with high CD4+ T cells counts (CD4 > 350/μl, *n* = 10) prior to cART had higher expression of cellular factors in JAK-STAT pathway than that of subjects with low CD4 + T cells count (CD4 < 350/μl, *n* = 8), especially for IRF-3/7/9, TLR-1/4/6/8, OAS-1, MxA, PKR, Tetherin, and STAT-2 (*p* < 0.05) (Figure 5).

## DISCUSSION

In the present study, we examined the *in vivo* impact of HIV-1 infection on JAK-STAT signaling pathway-mediated anti-HIV-1 immunity. JAK-STAT signaling pathway has been implicated in TLR-mediated pathogen recognition, IRFs activation [26], the induction of IFN-inducible genes such as MxA and ISG56 [12, 22]. We found that HIV-1-infected subjects had lower levels of the TLRs, IRFs and the cellular anti-HIV factors (Figures 1, 2, 3). These findings indicate that HIV-1-infected subjects had a compromised JAK-STAT-mediated antiviral immunity. Interestingly, cART could only restore some of the HIV-1 restriction factors (Figure 3). We further analyzed the correlations between each two factors in JAK-STAT pathway, including adaptor molecular such as MyD88 and STAT-2. As shown in Figure 4 and Supplementary Table 1, significantly positive correlations were found in most factors in the control subjects, but only less than half of factors in HIV-1-infected subjects, and stably increased with the cART. These data were in agreement with the expression of TLRs, IRFs, and anti-HIV-1 factors in JAK-STAT pathway, indicating that cART can restore the impaired JAK-STAT pathway in PBMCs of HIV-1-infected subjects. While the mechanism(s) involved in HIV-1 infection-mediated impairment of JAK-STAT pathway remain to be determined, it has been shown that Vpr and Vif, two HIV-1 accessory proteins could bind to TANK-binding kinase 1 (TBK1) and inhibit TBK1 autophosphorylation [27], which is a key step in the activation of IFN and NF-κB signaling pathways. Also, HIV-1 Vpu accessory protein is known to induce IRF3 cleavage [28]. The HIV-1-infected subjects with high CD4+ T cells count (> 350/μl) appeared to respond better to cART in terms of the restoration of some of the antiviral factors in JAK-STAT pathway. These findings are clinically significant as they indicate that cART is beneficial for HIV-1-infected subjects who have CD4+ T cell counts > 350/μl than those with the counts < 350/μl. However, further studies with more clinical specimens are needed in order to determine clinical beneficial of early cART in the restriction of host innate immunity against HIV-1.

## MATERIALS AND METHODS

### Subjects

Eighteen HIV-1-infected subjects (17 males and one female) and 18 age-matched healthy donors (16 males and two females) were enrolled if they aged 18 or over and volunteered with written informed consent. The study was approved by the Ethics Committee of Wuhan Centers for Disease Prevention & Control. All HIV-1-infected subjects were examined by medical screening, and voluntarily received cART. The treatment regimen and algorithm TDF+3TC+EFV (17 patients) or AZT+3TC+NVP (1 patient) were carried out according to the China free cART manual [29], in which the threshold for cART eligibility was recommend as CD4+ T cells < 350/μL [30] and suggested therapy regimen was AZT/d4T+3TC+NVP/EFV.

### Blood sampling

For HIV-1-infected subjects, whole blood was collected prior to cART (0 month), 1 month, 3 months, and 6 months after cART. Estimation of absolute CD4+ T cell counts and plasma HIV-1 loads were tested as described previously [31]. Whole blood (10 mL) of HIV-1-infected subjects and healthy donors were subjected for the separation of PBMCs by standard Ficoll-Paque density gradient centrifugation, according to previously described technique [32, 33]. Total cellular nucleic acid was extracted from PBMCs using the TRI Reagent (Molecular Research Center, Clicinnati, OH) according to the manufacturer's protocol, and stored at −70°C for RT-PCR analysis.

### Real-time RT-PCR

Total RNA (1 μg) was reverse transcribed using the RT system (Promega, Madison, WI) for 1 h at 42°C. 1.5 μL cDNA was subjected as a template for real-time PCR quantification. Using the power SYBR^®^ Green PCR Master Mix (Life Technologies, California), messenger RNAs (mRNAs) in PBMCs were detected. The level of glyceraldehydes-3-phosphate dehydrogenase (GAPDH) mRNA was used as an endogenous reference to normalize the quantities of target mRNA. The primers used for the detection of the mRNAs (Table 2) were designed as previously described [12, 22]. The PCR results were analyzed by the 2^−ΔΔCT^ methods [34].

**Table 2:** Primers used for real-time RT-PCR

Primer	Orientation	Sequences
GAPDH	Sense	5′-GGTGGTCTCCTCTGACTTCAACA-3′
	Antisense	5′-GTTGCTGTAGCCAAATTCGTTGT-3′
TLR-1	Sense	5′-GCCTATATGCAAAGAGTTTGGC-3′
	Antisense	5′-CTCTCCTAAGACCAGCAAGACC-3′
TLR-4	Sense	5′-CAGAGTTTCCTGCAATGGATCA-3′
	Antisense	5′-GCTTATCTGAAGGTGTTGCACAT-3′
TLR-6	Sense	5′-ATTGAAAGCATTCGTGAAGAAG-3′
	Antisense	5′-ACGGTGTACAAAGCTGTCTGTG-3′
TLR-7	Sense	5′-AAAATGGTGTTTCCAATGTGG-3′
	Antisense	5′-GGCAGAGTTTTAGGAAACCATC-3′
TLR-8	Sense	5′-TTATGTGTTCCAGGAACTCAGAGAA-3′
	Antisense	5′-TAATACCCAAGTTGATAGTCGATAAGTTTG-3′
TLR-9	Sense	5′-TAGGACAACAGCAGATACTCCAGG-3′
	Antisense	5′-TACCAACATCCTGATGCTAGACTC-3′
IRF-3	Sense	5′-ACCACCCGTGGACCAAGAG-3′
	Antisense	5′-TACCAAGGCCCTGAGGCAC-3′
IRF-7	Sense	5′-TGGTCCTGGTGAAGCTGGAA-3′
	Antisense	5′-GATGTCGTCATAGAGGCTGTTGG-3′
IRF-9	Sense	5′-GCATCAGGCAGGGCACGCTGCACCCG-3′
	Antisense	5′-GCCTGCATGTTTCCAGGGAATCCGG-3′
OAS-1	Sense	5′-AGAAGGCAGCTCACGAAACC-3′
	Antisense	5′-CCACCACCCAAGTTTCCTGTA-3′
MxA	Sense	5′-GCCGGCTGTGGATATGCTA-3′
	Antisense	5′-TTTATCGAAACATCTGTGAAAGCAA-3′
A3G	Sense	5′-TCAGAGGACGGCATGAGACTTAC-3′
	Antisense	5′-AGCAGGACCCAGGTGTCATTG-3′
Tetherin	Sense	5′-AAGAAAGTGGAGGAGCTTGAGG-3′
	Antisense	5′-CCTGGTTTTCTCTTCTCAGTCG-3′
PKR	Sense	5′-AGAGTAACCGTTGGTGACATAACCT-3′
	Antisense	5′-GCAGCCTCTGCAGCTCTATGTT-3′
ISG-56	Sense	5′-TTCGGAGAAAGGCATTAGA-3′
	Antisense	5′-TCCAGGGCTTCATTCATAT-3′
IL-6	Sense	5′-AGGAGACTTGCCTGGTGAAA-3′
	Antisense	5′-CAGGGGTGGTTATTGCATCT-3′
IL-10	Sense	5′-CTT TAA TAA GCT CCA AGA GAA AGG C-3′
	Antisense	5′-CAG ATC CGA TTT TGG AGA CC-3′
MyD88	Sense	5′-CCGCGCTGGCGGAGGAGATGGAC-3′
	Antisense	5′-GCAGATGAAGGCATCGAAACGCTC-3′
STAT-2	Sense	5′-CCCCATCGACCCCTCATC-3′
	Antisense	5′-GAGTCTCACCAGCAGCCTTGT-3′

### Statistical analysis

Where appropriate, data were expressed as mean ± standard deviations (SD). For comparison of the means of two groups, statistical significance was assessed by Student's *t* paired (prior to versus after cART) or unpaired (HIV-1-infected subjects versus HIV-1-uninfected subjects) test. Statistical analyses were performed with GraphPad Instat statistical software (San Diego, CA). Statistical significance was defined as *p* < 0.05.

## SUPPLEMENTARY MATERIALS FIGURES AND TABLES





## References

[1] Hou W, Wang X, Ye L, Zhou L, Yang ZQ, Riedel E, Ho WZ (2009). Lambda interferon inhibits human immunodeficiency virus type 1 infection of macrophages. Journal of virology.

[2] Takeuchi O, Akira S (2009). Innate immunity to virus infection. Immunological reviews.

[3] Maheaswari R, Sivasankar K, Subbarayan S (2014). Toll gates: An emerging therapeutic target. Journal of Indian Society of Periodontology.

[4] Lu X, Wang J, Jin X, Huang Y, Zeng W, Zhu J (2015). IFN-CSP inhibiting hepatitis B virus in HepG2.2.15 cells involves JAK-STAT signal pathway. BioMed research international.

[5] Zhang Q, Wang Y, Wei L, Jiang D, Wang JH, Rao HY, Zhu L, Chen H, Fei R, Cong X (2008). Role of ISGF3 in modulating the anti-hepatitis B virus activity of interferon-alpha in vitro. Journal of gastroenterology and hepatology.

[6] Heim MH (2009). HCV innate immune responses. Viruses.

[7] Alexopoulou L, Holt AC, Medzhitov R, Flavell RA (2001). Recognition of double-stranded RNA and activation of NF-kappaB by Toll-like receptor 3. Nature.

[8] Heil F, Hemmi H, Hochrein H, Ampenberger F, Kirschning C, Akira S, Lipford G, Wagner H, Bauer S (2004). Species-specific recognition of single-stranded RNA via toll-like receptor 7 and 8. Science (New York, NY).

[9] DeCarlo CA, Rosa B, Jackson R, Niccoli S, Escott NG, Zehbe I (2012). Toll-like receptor transcriptome in the HPV-positive cervical cancer microenvironment. Clinical & developmental immunology.

[10] Janssens S, Beyaert R (2003). Role of Toll-like receptors in pathogen recognition. Clinical microbiology reviews.

[11] Sato M, Suemori H, Hata N, Asagiri M, Ogasawara K, Nakao K, Nakaya T, Katsuki M, Noguchi S, Tanaka N, Taniguchi T (2000). Distinct and essential roles of transcription factors IRF-3 and IRF-7 in response to viruses for IFN-alpha/beta gene induction. Immunity.

[12] Liu MQ, Zhou DJ, Wang X, Zhou W, Ye L, Li JL, Wang YZ, Ho WZ (2012). IFN-lambda3 inhibits HIV infection of macrophages through the JAK-STAT pathway. PLoS One.

[13] Au WC, Pitha PM (2001). Recruitment of multiple interferon regulatory factors and histone acetyltransferase to the transcriptionally active interferon a promoters. The Journal of biological chemistry.

[14] Pagliaccetti NE, Robek MD (2010). Interferon-λ in HCV Infection and Therapy. Viruses.

[15] Larrea E, Aldabe R, Molano E, Fernandez-Rodriguez CM, Ametzazurra A, Civeira MP, Prieto J (2006). Altered expression and activation of signal transducers and activators of transcription (STATs) in hepatitis C virus infection: in vivo and in vitro studies. Gut.

[16] Melen K, Fagerlund R, Nyqvist M, Keskinen P, Julkunen I (2004). Expression of hepatitis C virus core protein inhibits interferon-induced nuclear import of STATs. Journal of medical virology.

[17] Bode JG, Ludwig S, Ehrhardt C, Albrecht U, Erhardt A, Schaper F, Heinrich PC, Haussinger D (2003). IFN-alpha antagonistic activity of HCV core protein involves induction of suppressor of cytokine signaling-3. FASEB journal.

[18] Kawaguchi T, Yoshida T, Harada M, Hisamoto T, Nagao Y, Ide T, Taniguchi E, Kumemura H, Hanada S, Maeyama M, Baba S, Koga H, Kumashiro R (2004). Hepatitis C virus down-regulates insulin receptor substrates 1 and 2 through up-regulation of suppressor of cytokine signaling 3. The American journal of pathology.

[19] Weber F, Haller O (2007). Viral suppression of the interferon system. Biochimie.

[20] Randall RE, Goodbourn S (2008). Interferons and viruses: an interplay between induction, signalling, antiviral responses and virus countermeasures. The Journal of general virology.

[21] McMichael AJ, Borrow P, Tomaras GD, Goonetilleke N, Haynes BF (2010). The immune response during acute HIV-1 infection: clues for vaccine development. Nature reviews Immunology.

[22] Zhou Y, Wang X, Liu M, Hu Q, Song L, Ye L, Zhou D, Ho W (2010). A critical function of toll-like receptor-3 in the induction of anti-human immunodeficiency virus activities in macrophages. Immunology.

[23] Haile WB, Gavegnano C, Tao S, Jiang Y, Schinazi RF, Tyor WR (2016). The Janus kinase inhibitor ruxolitinib reduces HIV replication in human macrophages and ameliorates HIV encephalitis in a murine model. Neurobiology of disease.

[24] Akira S, Takeda K (2004). Toll-like receptor signalling. Nature reviews Immunology.

[25] Sato Y, Goto Y, Narita N, Hoon DS (2009). Cancer Cells Expressing Toll-like Receptors and the Tumor Microenvironment. Cancer microenvironment.

[26] Nilsen NJ, Vladimer GI, Stenvik J, Orning MP, Zeid-Kilani MV, Bugge M, Bergstroem B, Conlon J, Husebye H, Hise AG, Fitzgerald KA, Espevik T, Lien E (2015). A role for the adaptor proteins TRAM, TRIF in toll-like receptor 2 signaling. The Journal of biological chemistry.

[27] Harman AN, Nasr N, Feetham A, Galoyan A, Alshehri AA, Rambukwelle D, Botting RA, Hiener BM, Diefenbach E, Diefenbach RJ, Kim M, Mansell A, Cunningham AL (2015). HIV Blocks Interferon Induction in Human Dendritic cells and Macrophages by Dysregulation of TBK1. Journal of virology.

[28] Park SY, Waheed AA, Zhang ZR, Freed EO, Bonifacino JS (2014). HIV-1 Vpu accessory protein induces caspase-mediated cleavage of IRF3 transcription factor. The Journal of biological chemistry.

[29] Zhang FJ China free ART manual.

[30] Wang L, Ge L, Wang L, Morano JP, Guo W, Khoshnood K, Qin Q, Ding Z, Sun D, Liu X, Luo H, Tillman J, Cui Y (2015). Causes of Death among AIDS Patients after Introduction of Free Combination Antiretroviral Therapy (cART) in Three Chinese Provinces, 2010–2011. PLoS One.

[31] Liu MQ, Tang L, Kong WH, Zhu ZR, Peng JS, Wang X, Yao ZZ, Schilling R, Zhou W (2013). CD4+ T cell count, HIV-1 viral loads and demographic variables of newly identified patients with HIV infection in Wuhan, China. Journal of medical virology.

[32] Wang X, Tan N, Douglas SD, Zhang T, Wang YJ, Ho WZ (2005). Morphine inhibits CD8+ T cell-mediated, noncytolytic, anti-HIV activity in latently infected immune cells. Journal of leukocyte biology.

[33] Wang X, Ye L, Zhou Y, Liu MQ, Zhou DJ, Ho WZ (2011). Inhibition of anti-HIV microRNA expression: a mechanism for opioid-mediated enhancement of HIV infection of monocytes. The American journal of pathology.

[34] Livak KJ, Schmittgen TD (2001). Analysis of relative gene expression data using real-time quantitative PCR and the 2(−Delta Delta C(T)) Method. Methods (San Diego, Calif).

