# Supplementary material for: Combination antiretroviral therapy (cART) restores HIV-1 infection-mediated impairment of JAK-STAT signaling pathway
Source: Oncotarget. 2017 Feb 6;8(14):22524–33. doi: 10.18632/oncotarget.15121 (PMC5410242; doi:10.18632/oncotarget.15121)
Supplement: Supplementary file 1 [file oncotarget-08-22524-s001.pdf]

Combination antiretroviral therapy (cART) restores HIV-1 infection-mediated impairment of JAK-STAT signaling pathway

Supplementary Materials

Supplementary Table 1: *p* values for the correlations between each two factors in JAK-STAT pathway in PBMCs of healthy donors or HIV-1-infected subjects on cART. See Supplementary_Table_1
