# Supplementary material for: Combination antiretroviral therapy (cART) restores HIV-1 infection-mediated impairment of JAK-STAT signaling pathway
Source: Oncotarget. 2017 Feb 6;8(14):22524–33. doi: 10.18632/oncotarget.15121 (PMC5410242; doi:10.18632/oncotarget.15121)
Supplement: Supplementary file 2 [file oncotarget-08-22524-s002.doc]

**Supplementary Table 1:** p values for the correlations between each two factors in JAK-STAT pathway in PBMCs of healthy donors or HIV subjects on cART.

| **P values** | | **HIV-1-infected subjects prior to cART** | | | | | | | | | | | | | | | | |  |  |
| --- | --- | --- | --- | --- | --- | --- | --- | --- | --- | --- | --- | --- | --- | --- | --- | --- | --- | --- | --- | --- |
|  |  | **IRF-3** | **IRF-7** | **IRF-9** | **TLR-1** | **TLR-4** | **TLR-6** | **TLR-7** | **TLR-8** | **TLR-9** | **OAS-1** | **MxA** | **A3G** | **PKR** | **ISG56** | **Tetherin** | **IL-6** | **IL-10** | **MyD88** | **STAT-2** |
| **HIV-1-infected subjects on 1 mont s cART** | **IRF-3** |  | 0.3033 | 0.2466 | 0.9838 | **0.0335** | 0.4733 | 0.2953 | **0.037** | 0.5479 | **0.0226** | 0.1421 | **0.0003** | **0.0003** | 0.4184 | 0.2002 | 0.9903 | 0.3114 | 0.7478 | **0.007** |
|  | **IRF-7** | 0.3808 |  | **<0.0001** | **0.017** | 0.2002 | 0.1105 | **0.0245** | 0.8484 | **0.0245** | **0.0008** | **0.0003** | 0.4136 | 0.1573 | **0.0009** | **<0.0001** | **0.0107** | **0.0107** | 0.0778 | **0.0482** |
|  | **IRF-9** | 0.8484 | **0.0038** |  | **0.0032** | **0.037** | 0.1496 | 0.0644 | 0.5814 | **0.0273** | **0.0142** | 0.0861 | 0.081 | **0.017** | **0.0303** | **<0.0001** | **0.0327** | **0.0208** | 0.2538 | **0.0024** |
|  | **TLR-1** | 0.39 | **<0.0001** | **0.0399** |  | 0.823 | **0.0021** | **0.0196** | 0.5927 | **0.0045** | 0.5645 | 0.2096 | 0.7726 | 0.6867 | **0.0344** | **0.0319** | 0.0504 | **0.0065** | **0.0126** | 0.4836 |
|  | **TLR-4** | 0.4941 | 0.0827 | 0.559 | **0.0311** |  | 0.6273 | 0.4331 | 0.2033 | 0.3155 | **0.037** | 0.1445 | **0.0335** | **0.0017** | 0.2993 | **0.0287** | 0.5645 | 0.8804 | 0.3238 | **0.001** |
|  | **TLR-6** | 0.1126 | **0.0014** | 0.6687 | **<0.0001** | **0.0058** |  | 0.1168 | 0.717 | 0.261 | 0.7851 | 0.2226 | 0.5046 | 0.6987 | 0.2396 | 0.3854 | 0.261 | 0.3672 | 0.5207 | 0.7416 |
|  | **TLR-7** | 0.5207 | **0.0122** | 0.2875 | **0.0063** | 0.2647 | **0.0063** |  | **0.0072** | **0.0014** | **0.0118** | **0.0009** | 0.2836 | 0.2361 | **0.0083** | **0.0115** | **0.0058** | 0.2466 | **0.0232** | 0.5479 |
|  | **TLR-8** | 0.8357 | 0.095 | 0.9643 | 0.3583 | **0.0052** | 0.0644 | **0.0471** |  | 0.1396 | 0.1547 | 0.0565 | **0.0408** | **0.0273** | 0.6508 | 0.4888 | 0.8612 | 0.8676 | 0.804 | 0.8676 |
|  | **TLR-9** | 0.4184 | **0.028** | 0.8676 | **0.028** | 0.7231 | **0.0134** | **0.0011** | 0.2836 |  | **0.0361** | **0.0262** | 0.6627 | 0.1881 | **0.0142** | **0.0025** | **0.002** | **0.0036** | **0.0019** | 0.2993 |
|  | **OAS-1** | 0.5207 | 0.1547 | 0.1822 | 0.1881 | 0.6099 | 0.1126 | **0.0226** | 0.5645 | 0.2128 |  | **0.0009** | **0.0408** | **0.0033** | 0.0968 | **<0.0001** | **0.017** | 0.2161 | 0.0913 | **0.0327** |
|  | **MxA** | 0.4184 | **0.0063** | 0.2161 | 0.1147 | 0.0746 | 0.0968 | **0.0077** | **0.0028** | 0.0827 | **0.0138** |  | 0.39 | 0.2161 | **0.0015** | **0.003** | **0.0439** | 0.1765 | 0.2161 | 0.3717 |
|  | **A3G** | 0.2953 | 0.2684 | 0.119 | 0.5701 | **0.0439** | 0.7354 | 0.3322 | **0.0029** | 0.717 | 0.5701 | **0.0186** |  | **0.0006** | 0.7293 | 0.2361 | 0.4784 | 0.276 | 0.7851 | 0.0701 |
|  | **PKR** | 0.1971 | 0.4429 | **0.0232** | 0.5534 | 0.5534 | 0.9903 | 0.3451 | 0.4733 | 0.438 | **0.0303** | 0.2065 | **0.0303** |  | 0.9126 | **0.0138** | 0.5479 | 0.9514 | 0.9062 | **0.0072** |
|  | **ISG56** | 0.9579 | **0.0335** | 0.5927 | **0.0037** | 0.7109 | **0.0327** | 0.0399 | 0.7664 | **0.0118** | 0.147 | **0.0471** | 0.7851 | 0.6042 |  | **0.0058** | **0.018** | 0.059 | 0.1681 | 0.1302 |
|  | **Tetherin** | 0.9255 | **0.0353** | **0.0018** | **0.0399** | 0.1168 | 0.1881 | 0.2002 | 0.9449 | 0.7664 | **0.0018** | 0.1234 | 0.4836 | **0.0327** | 0.3073 |  | **0.0088** | **0.0043** | 0.658 | **0.0043** |
|  | **IL-6** | 0.6746 | 0.0686 | 0.9449 | 0.1851 | 0.3994 | 0.16 | **0.0122** | 0.7851 | **0.0017** | 0.1851 | **0.0361** | 0.5985 | 0.2647 | **0.0047** | 0.874 |  | 0.119 | **0.0058** | 0.51 |
|  | **IL-10** | 0.0686 | **0.018** | 0.6627 | **0.0344** | 0.932 | **0.0449** | 0.4331 | 0.9062 | **0.0026** | 0.3854 | 0.9968 | 0.328 | 0.0701 | **0.0493** | 0.7478 | **0.0399** |  | **0.0026** | 0.5046 |
|  | **MyD88** | 0.1212 | **0.0067** | 0.4888 | **0.0311** | 0.9773 | 0.0552 | 0.3495 | 0.9384 | **0.0054** | 0.3538 | 0.8548 | 0.438 | 0.1302 | **0.046** | 0.7416 | **0.0287** | **<0.0001** |  | 0.7478 |
|  | **STAT-2** | 0.8357 | **0.0018** | **<0.0001** | **0.0072** | **0.013** | 0.1822 | 0.1911 | 0.2836 | 0.9708 | 0.119 | **0.0399** | **0.0252** | **0.0202** | 0.4529 | **0.0003** | 0.6806 | 0.9384 | 0.8997 |  |

**Supplementary Table 1 (**continued)

| **P values** | | **HIV-1-infected subjects on 3 months cART** | | | | | | | | | | | | | | | | |  |  |
| --- | --- | --- | --- | --- | --- | --- | --- | --- | --- | --- | --- | --- | --- | --- | --- | --- | --- | --- | --- | --- |
|  |  | **IRF-3** | **IRF-7** | **IRF-9** | **TLR-1** | **TLR-4** | **TLR-6** | **TLR-7** | **TLR-8** | **TLR-9** | **OAS-1** | **MxA** | **A3G** | **PKR** | **ISG56** | **Tetherin** | **IL-6** | **IL-10** | **MyD88** | **STAT-2** |
| **HIV-1-infected subjects on six months cART** | **IRF-3** |  | **0.0311** | **0.0018** | 0.5207 | **0.038** | 0.3854 | 0.6627 | 0.2128 | 0.4089 | **0.0399** | **0.0034** | 0.0987 | 0.8612 | 0.1065 | **0.0449** | 0.9643 | **0.0418** | 0.3073 | **0.0037** |
|  | **IRF-7** | 0.095 |  | **0.0118** | **0.0134** | **<0.0001** | **0.0067** | **0.0165** | **0.005** | 0.226 | **<0.0001** | **<0.0001** | 0.5315 | 0.2466 | **0.0004** | **0.0014** | 0.8997 | 0.2953 | 0.1881 | **<0.0001** |
|  | **IRF-9** | **0.017** | **0.0013** |  | 0.095 | **0.0428** | 0.0987 | **0.0295** | **0.0122** | 0.7109 | **0.0021** | **<0.0001** | 0.1372 | 0.054 | **0.0085** | **0.0101** | 0.0913 | 0.4529 | 0.3627 | **<0.0001** |
|  | **TLR-1** | **0.027** | **0.0003** | **0.0147** |  | **0.0008** | **<0.0001** | **0.0004** | **0.0001** | **0.0037** | **0.0408** | **0.018** | 0.9255 | 0.0913 | **0.007** | **0.0196** | 0.6332 | 0.932 | **0.0126** | 0.0504 |
|  | **TLR-4** | **0.037** | **<0.0001** | **0.0226** | **<0.0001** |  | **0.0007** | **0.0147** | **<0.0001** | 0.3033 | **0.0067** | **0.0005** | 0.51 | 0.0701 | **0.0191** | **0.0002** | 0.9708 | 0.2193 | 0.2096 | **0.0009** |
|  | **TLR-6** | **0.014** | **<0.0001** | **0.0027** | **<0.0001** | **<0.0001** |  | **0.0003** | **0.0003** | **0.0025** | **0.0303** | **0.016** | 0.7851 | 0.1521 | **0.0063** | **0.0202** | 0.5153 | 0.932 | **0.0048** | **0.0232** |
|  | **TLR-7** | 0.1325 | **0.0022** | **0.0011** | **0.0029** | **0.0142** | **0.0017** |  | **0.0007** | **0.0094** | **0.0011** | **0.0037** | 0.3073 | 0.0762 | **0.0439** | **0.0047** | 0.1025 | 0.639 | **0.0004** | **0.0054** |
|  | **TLR-8** | **0.012** | **0.0011** | 0.1256 | **0.0002** | **<0.0001** | **0.0003** | **0.0428** |  | 0.2993 | **0.013** | **0.003** | 0.1736 | **0.0014** | 0.0861 | **0.0002** | 0.8293 | 0.1881 | 0.16 | **0.0027** |
|  | **TLR-9** | 0.4836 | **0.0016** | 0.1168 | **0.0043** | **0.0033** | **0.0142** | **0.0088** | **0.001** |  | 0.2128 | 0.3538 | 0.147 | 0.8612 | **0.0072** | 0.8357 | 0.2684 | **0.0418** | **0.0004** | 0.4041 |
|  | **OAS-1** | 0.0778 | **<0.0001** | **0.0048** | **0.0013** | **<0.0001** | **0.0002** | **0.0003** | **0.0122** | **0.0018** |  | **<0.0001** | 0.1085 | 0.3238 | **0.001** | **0.0013** | 0.458 | 0.3451 | 0.0968 | **<0.0001** |
|  | **MxA** | 0.147 | **<0.0001** | **0.0085** | **0.0013** | **0.0001** | **0.0005** | **0.0004** | **0.0009** | **0.0009** | **<0.0001** |  | 0.1234 | 0.1382 | **0.0013** | **0.0004** | 0.4941 | 0.1547 | 0.2361 | **<0.0001** |
|  | **A3G** | **0.005** | **0.0088** | **0.0021** | **0.0005** | **0.0063** | **0.0008** | **0.0052** | **0.0003** | **0.0052** | **0.0104** | **0.0107** |  | 0.4041 | 0.6927 | 0.0731 | 0.8484 | **0.0191** | 0.4993 | 0.1573 |
|  | **PKR** | 0.0617 | 0.6157 | 0.0861 | 0.2798 | 0.3583 | 0.0861 | **0.0361** | 0.2836 | 0.4136 | 0.2361 | 0.3808 | 0.0577 |  | 0.9643 | **0.0311** | 0.7914 | 0.3033 | 0.51 | 0.1681 |
|  | **ISG56** | 0.4993 | **0.0008** | 0.1006 | **0.0361** | **0.0097** | **0.022** | **0.0115** | **0.0097** | **0.0202** | **0.007** | **<0.0001** | 0.0778 | 0.6508 |  | 0.081 | 0.3717 | 0.3627 | **0.0311** | **0.0011** |
|  | **Tetherin** | **0.018** | **<0.0001** | **0.0003** | **0.0008** | **0.0012** | **<0.0001** | **0.0015** | **0.0428** | 0.0552 | **<0.0001** | **0.0006** | **0.0126** | 0.1765 | **0.0151** |  | 0.2993 | 0.1496 | 0.2293 | **0.0017** |
|  | **IL-6** | 0.6627 | 0.8293 | 0.0644 | 0.7851 | 0.6215 | 0.874 | 0.1168 | 0.2953 | 0.4836 | 0.7789 | 0.7478 | 0.8869 | 0.8421 | 0.6806 | 0.5479 |  | **0.0493** | **0.0295** | 0.6508 |
|  | **IL-10** | 0.0861 | 0.7602 | 0.8357 | 0.1941 | 0.1445 | 0.0931 | 0.261 | 0.0672 | 0.3947 | 0.4784 | 0.8612 | **0.0439** | 0.0565 | 0.639 | 0.6273 | 0.0672 |  | 0.0778 | 0.2193 |
|  | **MyD88** | **0.031** | 0.8869 | 0.823 | 0.6332 | 0.3155 | 0.51 | 0.5985 | **0.0471** | 0.8869 | 0.8484 | 0.8612 | 0.0878 | 0.6687 | 0.4784 | 0.7231 | 0.0746 | **0.0091** |  | 0.1911 |
|  | **STAT-2** | 0.0552 | **<0.0001** | **0.0001** | **0.0008** | **<0.0001** | **<0.0001** | **0.0003** | **0.0111** | **0.0156** | **<0.0001** | **<0.0001** | **0.0048** | 0.1445 | **0.0037** | **<0.0001** | 0.51 | 0.4479 | 0.823 |  |

**Supplementary Table 1 (**continued)

| **P values** | **Control group** | | | | | | | | | | | | | | | | | | |
| --- | --- | --- | --- | --- | --- | --- | --- | --- | --- | --- | --- | --- | --- | --- | --- | --- | --- | --- | --- |
|  | **IRF-3** | **IRF-7** | **IRF-9** | **TLR-1** | **TLR-4** | **TLR-6** | **TLR-7** | **TLR-8** | **TLR-9** | **OAS-1** | **MxA** | **A3G** | **PKR** | **ISG56** | **Tetherin** | **IL-6** | **IL-10** | **MyD88** | **STAT-2** |
| **IRF-3** |  | **0.0165** | **0.0361** | **0.0047** | **0.007** | **0.0029** | **0.0013** | **0.0042** | **0.0138** | **0.0006** | **0.0028** | **<0.0001** | **0.0065** | **0.0002** | **0.004** | 0.0516 | 0.1056 | 0.0746 | **0.0034** |
| **IRF-7** |  |  | **<0.0001** | **0.0004** | **0.0007** | **0.0001** | **<0.0001** | **0.0013** | **0.0015** | **<0.0001** | **<0.0001** | **0.0001** | **0.005** | **0.0002** | **<0.0001** | **0.0001** | **0.0004** | **<0.0001** | **<0.0001** |
| **IRF-9** |  |  |  | **0.0026** | **0.0118** | **0.0027** | **0.0027** | **0.0067** | **0.0245** | **0.0025** | **0.0008** | **0.0011** | **0.0196** | **0.0016** | **0.0001** | **0.003** | **0.0003** | **0.004** | **0.0008** |
| **TLR-1** |  |  |  |  | **<0.0001** | **<0.0001** | **<0.0001** | **<0.0001** | **<0.0001** | **<0.0001** | **<0.0001** | **<0.0001** | **<0.0001** | **<0.0001** | **<0.0001** | **0.0001** | **<0.0001** | **0.005** | **<0.0001** |
| **TLR-4** |  |  |  |  |  | **<0.0001** | **<0.0001** | **<0.0001** | **0.0006** | **<0.0001** | **<0.0001** | **<0.0001** | **<0.0001** | **0.0006** | **<0.0001** | **<0.0001** | **0.0002** | **0.0191** | **<0.0001** |
| **TLR-6** |  |  |  |  |  |  | **<0.0001** | **<0.0001** | **<0.0001** | **<0.0001** | **<0.0001** | **<0.0001** | **<0.0001** | **0.0001** | **<0.0001** | **<0.0001** | **0.0003** | **0.002** | **<0.0001** |
| **TLR-7** |  |  |  |  |  |  |  | **<0.0001** | **<0.0001** | **<0.0001** | **<0.0001** | **<0.0001** | **<0.0001** | **<0.0001** | **<0.0001** | **0.0001** | **0.0004** | **0.008** | **<0.0001** |
| **TLR-8** |  |  |  |  |  |  |  |  | **0.0001** | **<0.0001** | **<0.0001** | **<0.0001** | **<0.0001** | **0.0008** | **<0.0001** | **<0.0001** | **0.0007** | **0.0311** | **<0.0001** |
| **TLR-9** |  |  |  |  |  |  |  |  |  | **0.0003** | **<0.0001** | **0.0003** | **<0.0001** | **0.0023** | **<0.0001** | **0.0012** | **0.0191** | **0.0026** | **0.0002** |
| **OAS-1** |  |  |  |  |  |  |  |  |  |  | **<0.0001** | **<0.0001** | **<0.0001** | **<0.0001** | **<0.0001** | **0.0002** | **0.0017** | **0.006** | **<0.0001** |
| **MxA** |  |  |  |  |  |  |  |  |  |  |  | **<0.0001** | **<0.0001** | **<0.0001** | **<0.0001** | **0.0002** | **0.0001** | **0.0048** | **<0.0001** |
| **A3G** |  |  |  |  |  |  |  |  |  |  |  |  | **<0.0001** | **<0.0001** | **<0.0001** | **0.0002** | **0.0005** | **0.0023** | **<0.0001** |
| **PKR** |  |  |  |  |  |  |  |  |  |  |  |  |  | **0.0021** | **0.0002** | **0.0001** | **0.0008** | **0.0295** | **0.0002** |
| **ISG56** |  |  |  |  |  |  |  |  |  |  |  |  |  |  | **<0.0001** | **0.0126** | **0.0027** | **0.0142** | **<0.0001** |
| **Tetherin** |  |  |  |  |  |  |  |  |  |  |  |  |  |  |  | **<0.0001** | **0.0003** | **<0.0001** | **<0.0001** |
| **IL-6** |  |  |  |  |  |  |  |  |  |  |  |  |  |  |  |  | **0.0002** | **0.0005** | **<0.0001** |
| **IL-10** |  |  |  |  |  |  |  |  |  |  |  |  |  |  |  |  |  | **0.0025** | **0.0003** |
| **MyD88** |  |  |  |  |  |  |  |  |  |  |  |  |  |  |  |  |  |  | **0.0001** |
| **STAT-2** |  |  |  |  |  |  |  |  |  |  |  |  |  |  |  |  |  |  |  |
